# Effect of Music Practice on Anxiety and Depression of Iranian Dental Students

**Published:** 2017-05

**Authors:** Mahmood Ghasemi, Hana Lotfollahzadeh, Tahereh Kermani-Ranjbar, Mohammad Javad Kharazifard

**Affiliations:** 1 Associate Professor, Department of Periodontics, Dental Branch, Islamic Azad University, Tehran, Iran; 2 Dentist, Private Practice, Tehran, Iran; 3 Psychiatrist, Assistant Professor, School of Paramedical Sciences, Shahid Beheshti University of Medical Sciences, Tehran, Iran; 4 Epidemiologist, Dental Research Center, Dentistry Research Institute, Tehran University of Medical Sciences, Tehran, Iran

**Keywords:** Anxiety, Depression, Music Therapy, Students, Dental, Education, Dental

## Abstract

**Objectives:**

The practice of dentistry has long been associated with high levels of occupational stress and anxiety and music has been shown as a method of reducing stress. Considering the reportedly high level of stress among dental students and its consequences and also considering the positive effect of music therapy, the aim of this study was to evaluate the relationship between music practice and level of stress in dental students.

**Materials and Methods:**

In this analytical, cross-sectional study, 88 students, including 44 with a history of music practice and 44 matched controls without music practice who met the defined inclusion criteria, participated. Upon obtaining written informed consent, all volunteers filled the Beck anxiety inventory (BAI) and Beck depression inventory (BDI) questionnaires. Data were analyzed using the Kolmogorov-Smirnov test, and multiple linear regression test with backward method was used to evaluate the effect of demographic factors on anxiety and depression scores.

**Results:**

The level of anxiety was higher in students who did not have music practice and this difference was significant (P<0.001). The same was observed for depression (P=0.027). Other factors including age, gender, and being far from family had no significant effect on depression and anxiety (P>0.05). But level of anxiety and depression was higher in students of universities with tuition fee compared to free public institutes (P<0.05).

**Conclusions:**

It may be concluded that music practice can reduce anxiety and depression of dental students.

## INTRODUCTION

Stress is defined as pressure or worry caused by problems in life [1]. Stress has turned into an undeniably famous subject of study because it can cause both physical and mental ailments [2]. It has been reported that dental education creates significant anxiety in dental students [3,4] and dental students are under greater stress than the overall public [5, 6] having higher levels of sadness and are more impulsive than the age-matched controls [7, 8]. Hermanson [9] reported that mood disorders ranked third among conditions commonly seen in dental clinicians [9] and Cooper et al, [10] revealed that dental carrier is the most stressful health related profession. Both dental education and practice are stressful and can have negative impacts on individual prosperity [11, 12]. Many studies have assessed stress reduction programs for dental students. For instance, Schwartz et al, [13] designed comprehensive at home stress-reduction programs. Tisdelle et al, [14] assessed an anxiety program comprising of six 60-to 90-minute sessions of stress administration procedures (e.g., dynamic muscle leisure). They were allowed to routinely use these methods in between the sessions, and they were given homework assignments as well. Students in both groups showed diminished overall anxiety and an increase in overall quality of life. Howard et al, [15] compared two methods of stress reduction namely a synchro-energizer, goggles with glimmering lights and earphones playing musical sounds that would reportedly help the brain unwind, and dynamic muscle unwinding. Malathi and Damodaran [16] indicated significant reductions in anxiety after yoga practice by medical students and found that students in the yoga practice group performed better on exams than the control group. Aside from yoga, music practice can have optimal mental and physical impacts [17, 18]. Thus, music practice may be able to help alleviate some psychological conditions [2, 19, 20]. Evidence shows that music practice is effective for restlessness, nervousness and anxiety [21–23]. To our knowledge, there is no study to investigate the effect of music practice by students themselves on their anxiety level and therefore, the aim of this study was to evaluate the relationship between music practice and level of anxiety and depression of dental students.

## MATERIALS AND METHODS

In this analytical, cross-sectional study, 88 dental students including 44 with reported history of music practice and 44 matched subjects not playing any musical instrument from five dental schools (two tuition fee and three free public schools) in the city of Tehran, Iran were enrolled. The study was approved by the Research Committee of Dental School, Islamic Azad University, Tehran, Iran (Ref. No: 24337). Using Minitab software and α=0.05, β=0.2, P1=0.4 and P2=0, minimum sample size was determined to be 40 students in each group. Students who reported to have music practice for at least the past one year (n=44) were enrolled and accordingly students not playing any musical instrument (n=44) were also included in the study.

After obtaining informed consent forms, the students were asked about their demographic data including age, sex, dental school, marital status, living far from family (from the beginning of their dental education) and family history of depression or anxiety.

All subjects completed the Beck Anxiety Inventory (BAI) [24] and the Beck Depression Inventory (BDI) [25] questionnaires. The BAI had 21 questions of four items with score range of zero to three points (total score of zero to 63 points). The subjects were categorized into minimum (0<BAI<7), mild (8<BAI<15), moderate (16<BAI<25) and severe (26<BAI<63). The BDI had also 21 questions of four items with score range of zero to three points (total score of zero to 63 points). The subjects were categorized into normal (1<BDI<10), mild depression (11<BDI<16), requiring psychiatric consultation (17<BDI<20), moderate depression (21<BDI<30), severe depression (31<BDI<40), and very severe depression (BDI > 40). The validity and reliability of the Persian version of these questionnaires has been previously confirmed in the Iranian population [26,27].

Data analysis was performed using SPSS 21 (SPSS Inc., IL, USA). One-sample Kolmogorov-Smirnov test was used to check the normal distribution of data. Multiple linear regression test with backward method was used to evaluate the effects of demographic factors on anxiety and depression scores. P values less than 0.05 were considered significant.

## RESULTS

The subjects included 50 males and 38 females with a mean age of 23 years. Demographic information of the subjects is presented in Table 1 and Kolmogorov-Smirnov test showed that the data were normally distributed. The level of anxiety was significantly (P<0.001) higher (17.47±10.11 versus 10.72±8.33) and more severe in subjects without music practice (Table 2). The depression was significantly (P=0.027) higher (16.22±11.01 versus 11.47±8.68) and more severe in subjects without music activity (Table 3). Multiple linear regression analysis showed that among the studied contributing factors, only the university type showed a significant difference and students in two tuition-fee (Azad and Pardis) universities had higher anxiety level compared to the other three government dental schools (P< 0.05). The other factors including age, gender, and being far from family had no significant effect on depression and anxiety (P > 0.05; Table 4).

**Table 1.** Demographic characteristics of the two groups of Iranian dental students with (n=44) and without (n=44) history of music practice

**Music practice**	**No music practice**	**(n=44)**	**(n=44)**
**Age (years)**	Mean± standard deviation	23.34±1.79	23.25±1.55
**Sex**	Male	19	19
	Female	25	25
**University**	Azad	23	20
	Beheshti	6	6
	Tehran	6	7
	Shahed	5	6
	Pardis	4	5
**Living far from family**	Yes	6	7
	No	38	37
**Family history of depression**	Yes	4	5
	No	40	39

**Table 2.** Anxiety level of Iranian dental students with (n=44) and without (n=44) history of music practice

		**Music practice**	**No music practice**
**Anxiety**	**(Mean± S.D)**	10.73±8.33	17.47±10.11^*^
**Minimum**		16	11
**Mild**		7	12
**Moderate**		8	17
**Severe**		3	4

* Statistically significant difference

**Table 3.** Depression level of Iranian dental students with (n=44) and without (n=44) history of music practice

		**Music practice**	**No music practice**
**Depression**	**(Mean± standard deviation)**	11.47±8.68	16.23±11.01^*^
**Normal**		24	13
**Mild**		9	5
**Moderate**		9	16
**Severe**		2	9
**Very severe**		0	1

* Statistically significant difference

**Table 4.** P values of Regression analysis of contributing factors to anxiety and depression in the Iranian dental students with and without music practice

	**Anxiety**	**Depression**
**Sex**	0.186	0.925
**University**	0.0001^*^	0.532
**Music practice**	0.001^*^	0.027^*^
**Studying away from home**	0.158	0.439

* significant correlation at 0.05 level.

## DISCUSSION

The results of this study showed that anxiety and depression were higher and more severe in dental students without history of music practice.

It was also observed that students studying in tuition fee schools were more stressed out compared to those studying in the other three free public schools. Educational costs have also been reported as a contributing factor to stress and anxiety in previous studies [28]. Muirhead and Locker [28] and Morse and Dravo [29] in their studies on Canadian and Fijian dental students, respectively, found that the high tuition was the greatest source of stress. Morse and Dravo [29] also reported that stress levels were higher in private school students. Dental career is accompanied with high stress levels, and mental disorders are the third problem among dentists [9]. Some authors have claimed that it is the most stressful health-related profession [10]. The stress level is different according to the geographical location, college type, and students and it was higher in clinical courses and treatment times among Spanish and Greece students, respectively [14]. Given the problems associated with dental students’ anxiety, it is unfortunate that only a few studies have examined stress-management interventions.

Considering the negative impact of anxiety on the students themselves, their academic performance, their dental work, and their patients’ comfort level, it has been recommended that dental students be provided with a stress management program during their dental training [3].

Malathi and Damodaran [16] found the beneficial role of yoga in not only decreasing the anxiety level but also attenuating the increase in the anxiety score in stressful states such as exams for medical students. Shankarapillai et al, [30] also observed that yogic breathing had a significant effect on the reduction of state trait anxiety level of dental students.

Art-based therapies and interventions are used in diverse populations and age groups to help alleviate symptoms and induce therapeutic and psychosocial effects in a wide variety of serious chronic conditions, illnesses, mental disorders, etc. For example, pain, breathlessness, stiffness, stress, depression, fatigue, anxiety and other symptoms are mitigated by the use of art therapy [30] and music therapy [31]. For either a patient or a healthy individual, engagement with the arts also enhances one’s well-being and quality of life [32]. Alzahem et al [33] stated that despite high frequency of stress among dental students, there is a lack of comprehensive stress reduction program for them. However, music was reported as one of the effective methods for stress-reduction by the authors.

Ahmad et al, [22] also observed that music was a popular remedy for stress reduction in the second, fourth and fifth year dental students. Also, there are studies about the role of music for reduction of stress among patients [34,35]. To our knowledge. it is the only study to provide evidence for the beneficial effect of music practice on reducing anxiety and depression in dental students; this area requires more attention in stress management programs.

Although some of the findings of the present study are easier to interpret (for example age and gender), future research should address other demographic factors and their relation to the level of anxiety and depression of students. The socioeconomic background of the participants may limit the applicability of the results of this study to other institutions and further research is recommended in this respect.

## CONCLUSION

According to the results of the present study, it may be concluded that music practice can reduce the anxiety and depression of dental students. Our findings once again demonstrated the role of stress-reducing programs in the educational curriculum to decrease dental students’ anxiety and this in turn may increase their learning abilities.
